# Establishment of a fluorescent *in situ* hybridization assay for imaging hepatitis B virus nucleic acids in cell culture models

**DOI:** 10.1038/emi.2017.84

**Published:** 2017-11-08

**Authors:** Xiaonan Zhang, Lei Yue, Zhanqing Zhang, Zhenghong Yuan

**Affiliations:** 1 Research Unit, Shanghai Public Health Clinical Center, Fudan University, Shanghai 200032, China; 2 Key Laboratory of Medical Molecular Virology at School of Basic Medical Sciences, Shanghai Medical College, Fudan University, Shanghai 200032, China; 3 Department of Hepatology, Shanghai Public Health Clinical Center, Fudan University, Shanghai 200032, China

**Keywords:** cccDNA, 3D-SIM, 3D-STORM, FISH, HBV

## Abstract

While chronic hepatitis B remains a global public health problem, the detailed spatiotemporal dynamics of the key molecular events leading to the multiplication and egress of hepatitis B virus (HBV) are still largely unclear. Previously, we developed a chromogenic *in situ* hybridization assay for detection of HBV RNA, DNA and covalently closed circular DNA in clinical liver biopsies. Here, we report the establishment of a fluorescent *in situ* hybridization method for the visualization of HBV RNA, HBV core particle DNA and intranuclear DNA in a tetracycline-inducible HBV replication system (HepAD38) and a *de novo* infection system (HepG2-NTCP). Using 3D-STORM (three-dimensional stochastic optical reconstruction microscopy), we were able to obtain images of HBV RNA and DNA with improved spatial resolution allowing in-depth analyses of key virological events within complex subcellular compartments. Taken together, these techniques should facilitate a deeper understanding of the molecular events of the HBV life cycle and shed new light on the intricate mechanisms of virus–host interactions.

## INTRODUCTION

The hepatitis B virus (HBV) continues to be a global public health issue, with over 240 million chronically infected individuals worldwide. Chronic hepatitis B is linked to a high risk of developing liver fibrosis, cirrhosis and hepatocellular carcinoma, which result in over 780 000 deaths annually.^1^ HBV is a member of the hepadnaviridae family, a family of small, hepatotropic DNA viruses. HBV has a 3.2-kb, partially double-stranded genome. The covalently closed circular DNA (cccDNA) in the nucleus, which serves as the template for the transcription of pregenomic RNA (pgRNA) and other subgenomic RNAs (2.4, 2.1 and 0.7 kb RNA), is initially formed immediately after HBV enters and persists inside host cells.^2^ Viral minus-strand DNA synthesis takes place by reverse transcribing pgRNA encapsidated in core particles, followed by further polymerization of plus-strand DNA. Mature virions are assembled when capsid-packed relaxed circular DNA (rcDNA) is enveloped by HBV surface antigens and secreted into circulation. Concomitant gap filling, deproteinization and nuclear transport of rcDNA result in its transformation into cccDNA. This recycling process replenishes the HBV nuclear genetic reservoir and maintains its persistence.

In recent years, a wealth of knowledge regarding the way that HBV replicates and persists in infected hepatocytes has been gained. Much of it, however, has been obtained through analyses of biochemical fractionation and bulk measurements of viral proteins and nucleic acids. Detailed spatiotemporal information on viral nucleic acids in a highly organized tissue and subcellular context still awaits further examination. Previously, by modifying the ViewRNA *in situ* hybridization assay, we established a chromogenic assay for visualizing HBV RNA, DNA and cccDNA in liver specimens of chronic hepatitis B patients.^3^ However, a fluorescent *in situ* hybridization (FISH) method is needed when a high spatial resolution is required to analyze various steps in the viral life cycle.

Here, we demonstrate the development of a modified ViewRNA FISH method that permits spatial visualization of the major HBV nucleic acids (HBV RNAs, replicative intermediate and intranuclear DNA) in HepAD38 cells and in a HepG2-NTCP-based infection model. Enhanced resolution achieved by 3D-STORM (three-dimensional stochastic optical reconstruction microscopy) provided a finer view of virological events in the course of HBV infection.

## MATERIALS AND METHODS

### Cell culture and HBV infection

HepAD38, generously provided by Prof. Jutao Guo (Blumberg Institute, Doylestown, PA, USA), and HepG2-NTCP (A3 clone), a kind gift from Prof. Stephan Urban (University of Heidelberg, Heidelberg, Germany), were maintained in Dulbecco’s modified Eagle’s medium supplemented with 10% fetal calf serum, l-glutamine, penicillin and streptomycin (Gibco, Grand Island, NY, USA). All culture surfaces were precoated with rat tail collagen (BD Bioscience, Franklin Lakes, NJ, USA). HepAD38 cells were normally maintained in the presence of 1 μg/mL doxycycline (dox+) to switch off transcription of HBV pgRNA. To turn on robust HBV replication, cells were cultured in the absence of doxycycline (dox−) for at least seven days before cell fixation and FISH analysis. For FISH experiments, cells were seeded in collagen-coated four-well Lab-Tek chamber slides (Thermo Fisher, Waltham, MA, USA). Infection of HepG2-NTCP cells by HBV was performed essentially as described.^4^ Supernatants of HepAD38 cells (dox−) were collected and concentrated 100-fold by ultrafiltration (Amicon Ultra, 100 kDa; Millipore, Burlington, MA, USA). Concentrated supernatants were mixed with culture medium containing 4% PEG8000 (final concentration) and inoculated onto HepG2-NTCP cells for 4–12 h. The cells were subsequently washed three times with phosphate-buffered saline (PBS) and maintained in a culture medium supplemented with 2.5% dimethyl sulfoxide for five to seven days before fixation. The levels of secreted hepatitis B surface antigen (HBsAg) and hepatitis B e antigen (HBeAg), collected on days 5 and 7 postinfection, were routinely monitored by enzyme-linked immunosorbent assay.

### FISH assay

The FISH experiments for HBV RNA and DNA were performed using the QuantiGene ViewRNA ISH cell assay and accompanying probe sets (Panomics/Affymetrix, Fremont, CA, USA). The protocol was modified to allow simultaneous detection of HBV RNA and DNA. Briefly, HepAD38 or HepG2-NTCP cells infected with HBV were fixed with 3.7% formaldehyde in diethyl-pyrocarbonate-treated PBS for 30 min at room temperature. The fixative was then removed and the cells were washed with PBS, followed by a wash with 50% ethanol in nuclease-free water for 5 min and 70% ethanol for at least 1 h at 4 °C. Cells were rehydrated in PBS and permeabilized with detergent solution QC for 10 min. After washing with PBS, cells were digested with protease (provided in the kit) diluted 3000-fold with PBS and incubated at 37 °C for 10 min. After additional washing with PBS, cells were refixed with 3.7% formaldehyde for 5 min. In some experiments, to better visualize intranuclear HBV DNA, cells were treated with 0.1 n HCl for 5 min and rinsed with PBS. Before hybridization, the chamber on the slide was removed and the remaining liquid was decanted. Probe set 2 (VF6-16995, target region nt2959-837, minus strand) and Probe set 3 (VF1-20344, target region nt481-1541, plus-strand, reference sequence: AF100309) were diluted 100-fold, either alone or in combination, in Probe set diluent QF and applied to the slide, which was subsequently covered with a 24 × 50 mm^2^ coverslip and sealed with rubber cement. The sealed slide was preheated at 85 °C for 5 min and then 40 °C overnight in a Thermobrite hybridizer. Subsequent signal amplification was performed as described in the assay manual. The slide was finally mounted in Prolong Gold antifade mountant with 4',6-diamidino-2-phenylindole (Thermo Fisher). The fluorophore-conjugated label probes that hybridized to type-specific branched DNA (bDNA) were excited with 550 nm (type 1) and 650 nm (type 6) light, and images were captured using filter sets for Cy3 (red) and Cy5 (infrared red), respectively, and pseudocolored (red for type 1 probe and green for type 6 probe) throughout the manuscript.

### Microscopic analysis

Most of the images were taken on a Deltavision (GE Healthcare Life Sciences, Pittsburgh, PA, USA) epifluorescence microscope using a × 100 NA 1.4 oil-immersion objective and Photometrics CoolSNAP HQ2 CCD camera (Tucson, AZ, USA). Images were taken in a series of Z steps and processed with deconvolution. Some images were taken on a Zeiss Axio Imager Z2 microscope (Wetzlar, Germany) using a × 63 oil-immersion objective and a Hamamastu ORCA-Flash4.0 sCMOS camera (Hamamatsu City, Japan). Three-dimensional structured illumination (3D-SIM) imaging was performed using the Nikon 3D-SIM System (Tokyo, Japan) with a × 100 NA 1.4 oil-immersion objective (Nikon, Tokyo, Japan). Z-stack images (125 nm step size) were generated and were processed to generate Z-step videos and maximum intensity projections. STORM was performed on a Nikon N-STORM System (Tokyo, Japan) with a 3D reconstruction module. Briefly, HepAD38 cells (dox−) were seeded on a 35 mm dish with a glass coverslip bottom, and FISH assay was performed as described above using Probe set 2 only (type 6). Before STORM imaging, the sample was covered with imaging buffer (100 mM Tris (pH 8.0), 10% glucose, 100 mM 2-mercaptoethanol, 1.4 mg/mL glucose oxidase and 0.8 mg/mL catalase) and illuminated with a 647 nm laser (100% power). Stochastic images (10 000 frames) were recorded, and 3D-STORM images were reconstructed.

### Image and data analysis

Analyses of the images generated by epifluorescence, 3D-SIM and 3D-STORM imaging were carried out using NIS-Elements Ar 5.0 (Nikon). For each experimental condition, 20–30 images were taken randomly. In each image, the puncta in the Cy3 and Cy5 channels were counted and the spots/cell ratio was calculated. In addition, the area of the detected spots was measured. The irregularity of the spots was expressed using an index named ‘circularity’, which was calculated using the formula as follows: circularity=4 × *π* × area/perimeter^2^. Statistical analyses of the calculated data were performed and plotted using GraphPad Prism 6 (La Jolla, CA, USA).

## RESULTS

### Establishment of a FISH assay to visualize HBV RNA and DNA in HepAD38 cells

To test whether the ViewRNA methodology could also detect HBV DNA in addition to RNA, we first used HepAD38 cells as a model system, in which robust HBV replication can be switched on by removal of doxycycline.^5^ HepG2-NTCP cells were used as a negative control. The design of the probe sets was similar to our previous report.^3^ Probe set 2 (type 6) targeting the minus strand of the HBV genome was used to detect HBV replication intermediates. Probe set 3 (type 1), which had a target region that is 500 bp longer than that in the previous report, was used to visualize HBV major transcripts (pgRNA, 2.4/2.1 kb RNA and partially complementary to 0.7 kb RNA), although mature HBV DNA with long plus-strand DNA could also be detected.

**Figure 1:**
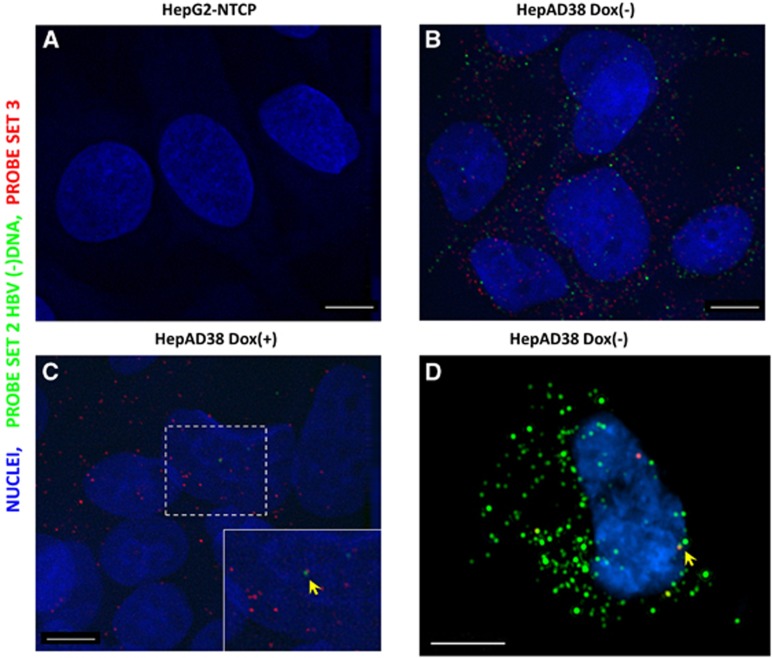
FISH of HBV RNA and DNA in a hepatoma cell line supporting HBV replication. HepG2-NTCP (**A**) and HepAD38 (**B**–**D**) cells, which are either maintained in doxycycline-supplemented medium (**C**) or doxycycline-free medium, were fixed and permeabilized, followed by hybridization with Probe set 2 and Probe set 3. After bDNA amplification, fluorophore-conjugated label probe was applied and images were acquired with a Deltavision epifluorescence microscope (**A**–**C**) and Nikon 3D-SIM microscope (Tokyo, Japan) (**D**). Scale bar, 4 μm.

While FISH performed on HepG2-NTCP cells yielded almost no spots (Figure 1A), HepAD38 (dox−) cells exhibited diffraction-limited dots in both red (mean 110.6, sd 36.0 dots per cell) and infrared channels (mean 53.8, sd 27.5 dots per cell), not only in the cytoplasm but also sparsely in nuclei (Figure 1B). In HepAD38 (dox+) cells, because of the shutdown of pgRNA transcription, HBV minus-strand DNA was rarely observed (mean 0.86, sd 1.3 dots per cell), while nuclear signals were occasionally found (Figure 1C, see the yellow arrow pointing to one red and one infrared spot in proximity). Nevertheless, as doxycycline does not shut down 2.4/2.1- and 0.7-kb transcripts, many spots were still found (median 33.5, sd 29.5 dots per cell) in the cytoplasm (Figure 1C). To further confirm that the nuclear signal observed under epifluorescence microscope was not an artifact caused by cytoplasmic signals in positions that were above or under the nuclei, we used 3D-SIM to acquire Z-stack images with an extended resolution. Indeed, we found that these signals were inside the nuclei (Figure 1D, see the yellow arrow, one red and one infrared spot in proximity). Supplementary Video 1 illustrates the Z-stack rendering of a typical result. It should be noted, however, that these signals may be derived from integrated viral DNA, cccDNA or protein-free rcDNA. Further differentiation of these types of molecules in HepAD38 cells is beyond the capability of the assay.

**Figure 2:**
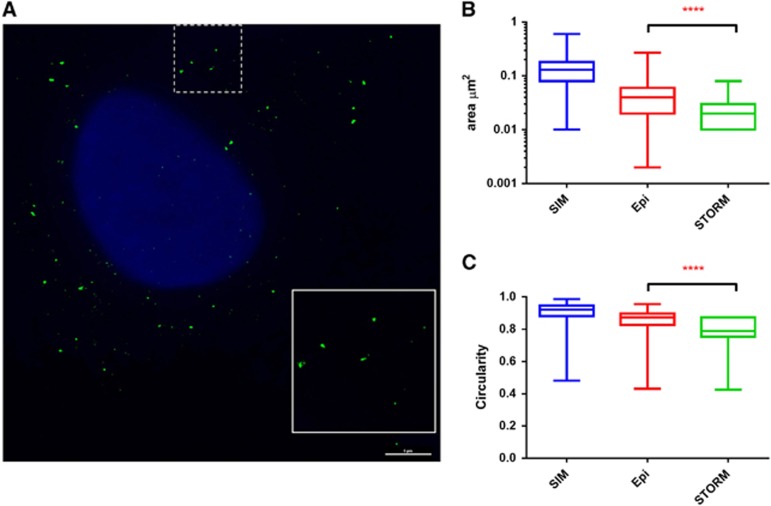
STORM imaging of HBV DNA. (**A**) HepAD38 cells cultured in doxycycline-free medium were fixed and permeabilized followed by hybridization with Probe set 2. After bDNA amplification, fluorophore-conjugated label probe was applied and images were reconstructed from stochastic images as described in the Materials and Methods section. Cell nuclei were imaged after STORM imaging using conventional fluorescence microscopy and merged. Scale bar, 5 μm. The surface areas (**B**) and circularity (**C**) of detected puncta in epifluorescence, 3D-SIM and 3D-STORM platforms were compared.

Since, theoretically, 3D-SIM can only provide a twofold improvement in resolution, we further attempted to use single molecule localization-based super-resolution microscopy, such as STORM, to obtain finer images. However, we observed that stochastic photo-switching was only achieved by the type 6 probe generated signal. As shown in Figure 2A, STORM reconstructed images in HepAD38 cells provided more details. In addition, we also generated 3D reconstituted images by the 3D-STORM method (Supplementary Video 2). Calculation of the areas of the detected spots from the three imaging platforms showed clear differences (Figure 2B). 3D-STORM generated signals that had an average area of 0.023 μm^2^ compared with 0.042 μm^2^ from images taken with the Deltavision epifluorescence microscope (Pittsburgh, PA, USA) (*P*<0.0001, Mann–Whitney test). The dots, as observed in epifluorescence and 3D-SIM (Figure 1), no longer had circular shapes (Figure 2A). The circularity index clearly indicated a reduction of regularity in 3D-STORM generated puncta (Figure 2C, *P*<0.0001, Mann–Whitney test). Unexpectedly, however, the dots detected by 3D-SIM showed the largest surface area and circularity in all three platforms, indicating a lack of resolution improvement. The intrinsic features in the lens, detectors and accompanying computation algorithms in the 3D-SIM system might be responsible for this phenomenon.

Overall, these results confirmed that *in situ* fluorescent detection of HBV RNA and DNA in both the cytoplasm and nuclei was technically feasible. We then tried to test this method on a *bona fide* HBV infection model.

### Visualization of viral nucleic acids in NTCP cells

**Figure 3:**
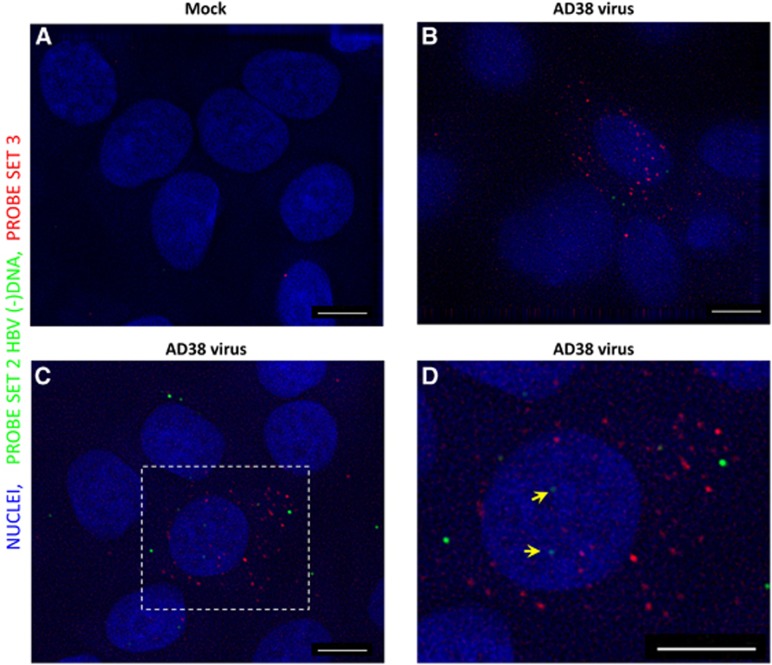
Detection of HBV RNA and DNA in HBV virus-infected HepG2-NTCP cells. HepG2-NTCP cells were either mock infected (**A**) or infected with 100 × concentrated HepAD38 cell supernatant (**B**–**D**). Seven days after infection, cells were fixed and permeabilized, followed by hybridization with Probe set 2 and Probe set 3. After bDNA amplification, fluorophore-conjugated label probe was applied and images were acquired using a Deltavision epifluorescence microscope with a × 100 oil-immersion objective. Image (**D**) was an enlarged area in the rectangle region of (**C**). Scale bar, 4 μm.

We next used a HBV-susceptible cell line, HepG2-NTCP (A3 clone), to test the performance of our methodology. As expected, FISH performed on mock-infected cells revealed almost no spots (Figure 3A). By contrast, ~38% of HBV-infected cells showed dots in both the red and infrared channels (Figures 3B and 3C). Many more puncta were observed following hybridization of Probe set 3 (mean 8.2, sd 12.2 dots per cell) compared with Probe set 2 (mean 4.3, sd 6.6 dots per cell, excluding FISH-negative cells), which suggested much higher amounts of HBV transcripts than cytoplasmic minus-strand DNA (Probe set 3/Probe set 2 ratio=1.9). We confirmed that the signals obtained were not derived from inoculated HBV nucleic acids but from active replication by including a control experiment in which UV-irradiated virus was mock-infected in parallel (Supplementary Figure 1B). Successful inactivation of the virus was ascertained by an enzyme-linked immunosorbent assay of HBsAg and HBeAg using supernatant collected 7 days after mock infection (data not shown). As expected, cells exposed to inactivated virus did not show a significant signal in contrast to the live-virus-infected group (Supplementary Figures 1C and 1D). Interestingly, we also found discernible spots within the nuclei of some infected HepG2-NTCP cells (Figure 3D, see the yellow arrows), which most likely indicated viral cccDNA. These results suggested that our assay was able to visualize the intranuclear DNA reservoir in a *bona fide* infection system.

## DISCUSSION

Studies on HBV biology provide an example of how molecular medicine has enhanced our understanding of the physical and chemical compositions of this virus as well as molecular details of its multiplication. The in-depth information that has been obtained has facilitated the development of prophylactic vaccines and chemical entities that lock the viral polymerase’s activity. These achievements have also led to a direct benefit to society by reducing the occurrence of infection and reversing the progression of liver disease.^1^ However, a complete cure for HBV is still a huge challenge that cannot be easily overcome without additional insights into the maintenance of viral persistence.^6^

In this study, we attempted to address this daunting problem from a distinct perspective, that is, to visualize and track major viral nucleic acids in a spatially resolved manner. This approach would allow us to probe some of the key questions, such as viral behavior at the single-cell level and subcellular dynamics of key molecular events (transcription, translation, encapsidation, reverse transcription and viral packaging, etc.). Indeed, two reports using FISH analysis to study hepatitis C virus (HCV) RNA have generated valuable insights into the key steps of HCV propagation^7^ and how the host’s innate sensing mechanism responds to viral multiplication.^8^ In addition, using bDNA-based FISH, Wieland *et al*^9^ successfully visualized HCV RNA and host mRNAs simultaneously in liver biopsies of hepatitis C patients and observed increased ISG expression in HCV RNA-positive cells. They also established a FISH assay based on ViewRNA technology to detect HBV RNA in formalin-fixed, paraffin-embedded and cryosections from HBV-infected liver tissue.^10^

The ViewRNA technology uses the bDNA signal amplification scheme, which was initially developed by the Chiron Corporation to quantify the HIV and HCV viral loads.^11, 12, 13, 14^ The bDNA scheme can also be used to quantify DNA molecules, such as HBV DNA, with a detection limit of 2000 copies.^15^ In addition, this technology has also been used for *in situ* detection of human papilloma virus, which also has a double-stranded circular DNA genome, in cell culture^16^ and in clinical tissue sections.^17^ Thus, it is reasonable to assume that HBV DNA of various forms could also be visualized using reagents in the ViewRNA assay. Compared with the assay reported by Calabrese *et al*,^10^ we used an additional mild heat denaturation treatment to aid in the hybridization with HBV DNA in double-stranded form. In addition, we pre-treated cells with hydrochloride to facilitate the relaxation of supercoiled cccDNA, making it more amenable for probe set annealing. It is worth noting that we also attempted to use single molecule FISH^18^ to visualize HBV RNA and DNA. However, our trials of single molecule FISH in HepAD38 and HepG2-NTCP cells had limited success (data not shown), possibly because of its intrinsic low signal-to-noise ratio. By contrast, the bDNA-based assay generated bright signals with negligible background, and images were more easily captured by conventional scientific digital cameras. Hence, we used the ViewRNA technology for further assay development.

We used probe sets targeting HBV minus-strand DNA and total RNA similar to our previous publication.^3^ However, it was not feasible to specifically target cccDNA by a gap-region-specific probe set as we have performed in liver biopsies because large amounts of protein-free rcDNA and rcDNA with almost complete plus-strand DNA are present in HepAD38 cells.^3, 5, 19^ This phenomenon would also likely be true in the HepG2-NTCP infection system. Thus, visualization of cccDNA solely depends on its intranuclear localization. Nevertheless, enough caution should be paid to the designation of these nuclear puncta as protein-linked or protein-free rcDNA with almost matured plus-strand DNA could also be detected when they are being processed and nuclear transported yet still not fully chromatinized.^20, 21^ Further experiments using costaining of key transcription and/or epigenetic components would validate the chromatinized nature of detected nuclear DNA.

Following a series of assay optimizations, we established the FISH method in the HepAD38 system and in a *de novo* infection system (HepG2-NTCP). Higher-resolution images of HBV DNA were obtained by STORM imaging, which detected much more irregularly shaped dots. It should be noted, however, that the bDNA amplification steps can generate a larger complex compared with the target itself. Thus, the contribution of the branched structure to the irregular shapes observed in STORM imaging should be further studied. Nevertheless, the decreased puncta size could help in resolving some finer structure in the context of host organelles or protein complexes.

In spite of the success of this new method, further improvement of the assay is needed and is under way as we could not achieve a high detection efficiency, possibly because of the double-stranded form of HBV rcDNA/cccDNA and its relatively weaker affinity to DNA probes compared with RNAs.

In summary, we successfully established a FISH assay for visualizing HBV nucleic acids (replicative intermediate and nuclear reservoir) in cell culture-based HBV models. In addition to diffraction-limited fluorescent imaging, we used the 3D-SIM and STORM imaging methods to achieve a higher resolution. The current FISH method would be useful for further dissecting the key molecular events in the propagation of HBV inside host cells and validating key epigenetic regulators of cccDNA maintenance and transcription.
