# Supplementary figures and images for: Establishment of a fluorescent in situ hybridization assay for imaging hepatitis B virus nucleic acids in cell culture models
Source: Emerg Microbes Infect. 2017 Nov 8;6(11):e98–. doi: 10.1038/emi.2017.84 (PMC5717087; doi:10.1038/emi.2017.84)

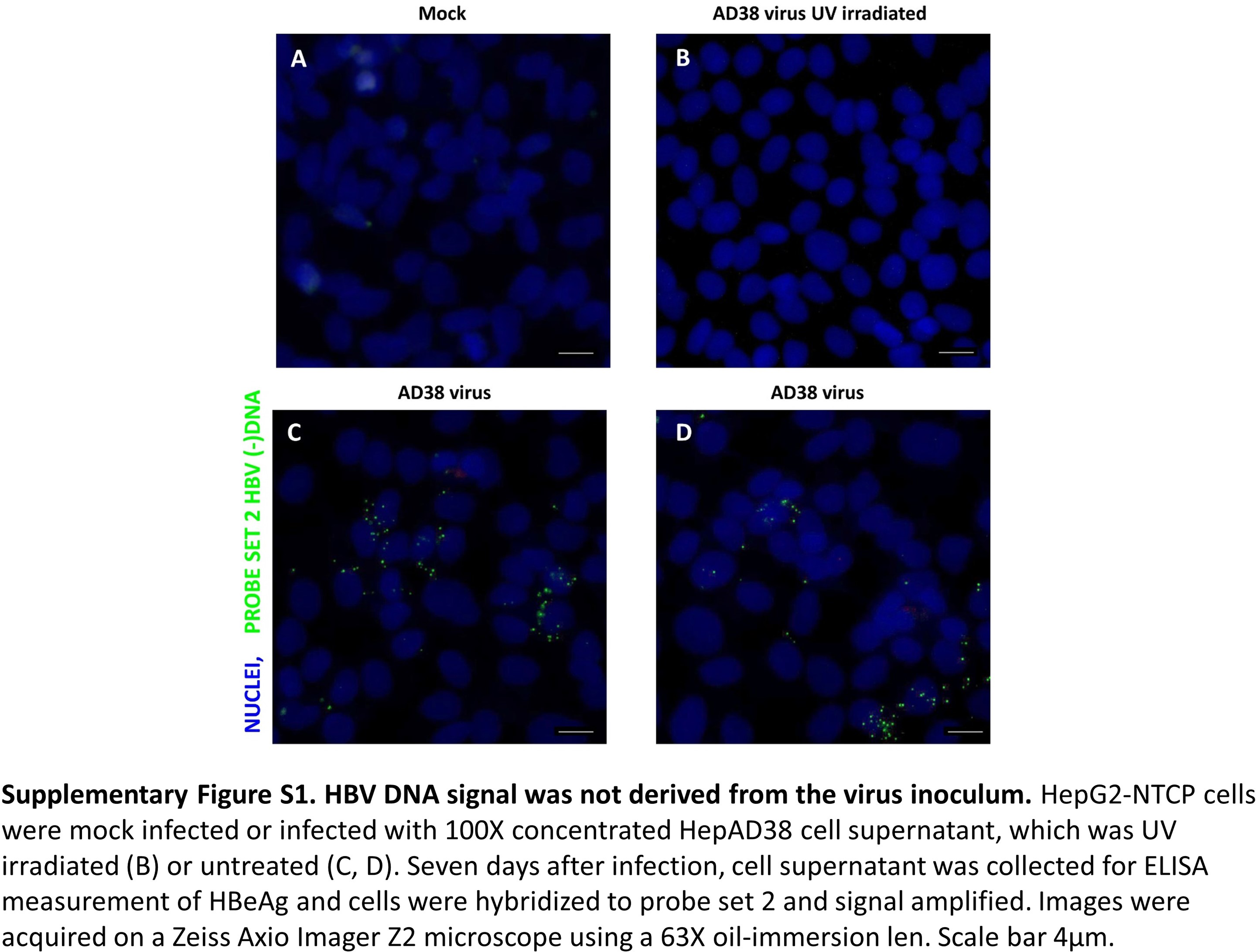

Supplement: Supplementary Figure S1 [file emi201784x1.tif]
